# Priority setting of ICU resources in an influenza pandemic: a qualitative study of the Canadian public's perspectives

**DOI:** 10.1186/1471-2458-12-241

**Published:** 2012-03-26

**Authors:** Diego S Silva, Jennifer L Gibson, Ann Robertson, Cécile M Bensimon, Sachin Sahni, Laena Maunula, Maxwell J Smith

**Affiliations:** 1 Dalla Lana School of Public Health, and Joint Centre for Bioethics, University of Toronto, 155 College Street, Suite 754, Toronto, ON, M5T 1P8, Canada; 2 Institute of Health Policy, Management, & Evaluation, and Joint Centre for Bioethics, University of Toronto, 155 College Street, Suite 754, Toronto, ON, M5T 1P8, Canada; 3 Dalla Lana School of Public Health, University of Toronto, 155 College Street, 6th Floor, Toronto, ON, M5T 3M7, Canada; 4 Joint Centre for Bioethics, University of Toronto, 155 College Street, Suite 754, Toronto, ON, M5T 1P8, Canada

**Keywords:** Priority setting, Pandemic influenza, Public perspectives, Qualitative methods, Ethics

## Abstract

**Background:**

Pandemic influenza may exacerbate existing scarcity of life-saving medical resources. As a result, decision-makers may be faced with making tough choices about who will receive care and who will have to wait or go without. Although previous studies have explored ethical issues in priority setting from the perspective of clinicians and policymakers, there has been little investigation into how the public views priority setting during a pandemic influenza, in particular related to intensive care resources.

**Methods:**

To bridge this gap, we conducted three public town hall meetings across Canada to explore Canadian's perspectives on this ethical challenge. Town hall discussions group discussions were digitally recorded, transcribed, and analyzed using thematic analysis.

**Results:**

Six interrelated themes emerged from the town hall discussions related to: ethical and empirical starting points for deliberation; criteria for setting priorities; pre-crisis planning; in-crisis decision-making; the need for public deliberation and input; and participants' deliberative struggle with the ethical issues.

**Conclusions:**

Our findings underscore the importance of public consultation in pandemic planning for sustaining public trust in a public health emergency. Participants appreciated the empirical and ethical uncertainty of decision-making in an influenza pandemic and demonstrated nuanced ethical reasoning about priority setting of intensive care resources in an influenza pandemic. Policymakers may benefit from a better understanding the public's empirical and ethical 'starting points' in developing effective pandemic plans.

## Background

An influenza pandemic has the potential to place considerable strain on health systems, forcing decision makers to set priorities for the use of available resources, including access to needed life-saving therapies. The impact that priority setting can have was evident during the 'mild pandemic' A(H1N1) in 2009-2010 during which some regional health systems in Canada and elsewhere experienced tremendous strain on local health resources, including access to life-saving resources. For example, the Winnipeg Regional Health Authority reported an unanticipated demand and subsequent shortage of sedation for ventilated H1N1-infected patients [1,2]. In health systems that are already strained by the impact of seasonal influenza [3-5] or other acute healthcare demands, an influenza pandemic may exacerbate pressure on hospitals and healthcare systems to competing health needs within limited resources.

Priority setting in an influenza pandemic can occur at a variety of levels, including the population level (e.g. priority groups for access to vaccination), the organizational level (e.g. health service priorities in hospitals), and the bedside (e.g. triage of patients for access to a ventilated intensive care bed). The allocation of scarce medical resources during an influenza pandemic requires careful consideration of the ethical values that underpin decision-making. From an ethics perspective, four key questions arise: i) who is entitled to a given health care resource?; ii) on what grounds ought a person have priority for access to a potentially life-saving resource over another person?; iii) how ought priority setting decisions to be made?; and iv) who ought to make these decisions?

Due to the potential life-saving nature of ventilated ICU beds, there have been a number of theoretical papers that have explored these questions, including the identification and weighing of different ethical values to guide their allocation during pandemics [6-13]. Some authors have argued that scarce intensive care resources should be allocated on the basis of who is the sickest (i.e., need);[6] some on the basis of age with priority given to the young (e.g., fair innings);[12] and others on the basis of who is most likely to survive or to benefit from treatment (i.e., medical utility) [7,14]. However, while there has been considerable debate among scholars, clinicians, and policymakers about how health resources, such as intensive care, should be allocated in a pandemic, there are few examples of investigation into or engagement with the public's perspectives on this issue [15].

Four reasons are commonly given for consulting the public on how resources should be allocated in an influenza pandemic. First, since the influenza virus is a community-acquired and transmitted infection that affects persons of all social and economic classes, it is truly a *public *disease. Hence, an effective public health response will depend on an informed and engaged public. Second, given the effects of influenza on healthcare systems and economies, public input about how health resources will be allocated is a key component of ensuring the democratic accountability of government policymakers [16-18]. Third, there is some evidence to suggest that public involvement can improve the quality of health policy decisions [19-23]. Finally, during a pandemic, when resources are scarce and not everyone may receive optimal treatment, trust is integral to public support for decisions and may help promote timely and effective responses. Thus, policymaking that is responsive to the public's experience and values may be essential for building public trust of political and social institutions in the face of an influenza pandemic. Given these considerations, the Canadian Program of Research on Ethics in a Pandemic (CanPREP), a research program based at the University of Toronto http://www.canprep.ca, conducted a national telephone survey and a series of public town hall meetings across Canada to elicit the public's perspectives on four ethical issues related to pandemic influenza - priority setting of health resources, the use of restrictive measures, healthcare worker duty to care, and global governance (i.e., government accountabilities for global management of pandemic crisis) - that had been identified previously in the University of Toronto Joint Centre for Bioethics' *Stand on Guard for Thee: Ethical Considerations in Preparedness Planning for Pandemic Influenza *report,[24,25] an influential document in pandemic planning ethics [13,26,27]. As this report had been developed with scholars, clinicians, and policymakers input and without public input, this public town hall meetings were also an opportunity to flesh out the range of values-based perspectives on these four ethical issues. In this paper, we present findings from three public town hall discussions about the ethical allocation of intensive care unit (ICU) beds during an influenza pandemic.

## Methods

### Study design

The CanPREP research team conducted a series of three public town hall meetings across Canada in order to probe the public's perspectives about ethical issues related to pandemic influenza. For the purpose of this study, we used the term 'public' to denote persons who are residents of Canada (e.g., citizens) and who are primarily healthcare system users (i.e., not primarily a healthcare provider, administrator, or policymaker). Each public town hall meeting was approximately eight hours in length and had the same format. After a plenary session on the epidemiology of influenza pandemics and an overview of the four ethical issues, participants were randomly sorted into four small groups of five-to-eight people to discuss and deliberate on a scenario illustrating one of the ethical issues (i.e., one small group per ethical issue in each town hall). At the end of the day, the four small groups reported back to the larger group on their scenario discussions. This paper reports only the results from the small group discussions about priority setting of health resources.

### Setting and participants

We conducted public three town halls in three major Canadian cities (Vancouver, British Columbia; Winnipeg, Manitoba; Saint John, New Brunswick). Overall, our aim was to recruit 25-40 people for each town hall meeting. Canadian residents aged 18 and over were recruited from the general public using local newspaper advertisements, online social networking sites (e.g. Facebook), and word-of-mouth by local study collaborators or through their local networks. Recruitment and town hall discussions were conducted in English. Participants were not paid for their participation, but were issued a travel voucher to cover the cost of parking, taxi fare, or public transit fares. The number of participants in each town hall was as follows: five in Vancouver, seven in Winnipeg, and five in Saint John for a total sample of 17 participants, of which 9 were female and 8 were male. No demographic information was solicited from the participants (e.g. ethnicity, income, etc.). However, based on self-reports during the initial round of introductions at the start of each small group discussion, the participants were primarily middle-aged and ranged in age from their early 20s to late 80s and divided equally among those who were currently employed and those who were retired or not employed. Several reported a history of community service work (e.g., as a community board member) or employment in the public sector. Although a few had a background in health care, most did not. With the exception of two participants, none had previous experience in pandemic planning; however, a few participants identified having experienced a community-based infectious outbreak (e.g., polio in the 1950s). Some participants reported having a current chronic health condition. The study received formal ethics approval from the Office of Research Ethics at the University of Toronto and all participants provided written consent prior to participation.

### Scenario

Participants were presented with a priority setting scenario involving resource scarcity in an intensive care unit and a series of discussion questions. The scenario was designed to introduce new information as the day progressed (referred to by team members as 'reveals') to see whether added details would change participants' opinions and arguments about the scenario. (For the scenario and reveals, please see Additional file 1 - Priority setting scenario.)

### Data collection & analysis

Small group discussions were facilitated by members of the CanPREP team. Small group discussions were audio recorded, transcribed verbatim, and verified by team members. We conducted a thematic analysis of transcripts within and across town halls using standard qualitative methods of analysis in order to identify convergences that cut across all three public town hall locations or divergences that were specific to particular town halls. The trustworthiness of our analysis was ensured by prolonged engagement with the data both individually and as a group, by meeting regularly as a team at each stage of analysis in order to discuss the interpretation of the results and consider the emerging themes, by presenting and discussing our findings to the larger CanPREP research team, and by keeping detailed team notes at each stage of analysis regarding what codes were added, removed, or collapsed in order to establish an "audit trail" [28].

## Results

Six interrelated themes emerged from our analysis of the data: ethical and empirical starting points; criteria for setting priorities; pre-crisis planning; in-crisis decision-making; the need for public deliberation and input; and participants' deliberative struggle with the ethical issues.

### Ethical & empirical starting points

Participants across all three town halls expressed what we are calling 'starting points' that seemed to function as guiding assumptions or 'givens' in their ensuing discussions. We use this term to capture a number of ideas or beliefs that participants invoked at different junctures, including assumptions about underlying moral values, such as equity, and ideas about 'the way things are'. Many participants began by describing the different reasons for which they agreed to participate in the town halls, often revealing personal stories related to public health emergencies caused by infectious diseases.

Well, I think I'm interested in the staff that are working in the hospitals and I think that came about from the fact that I was working as a nurse during the polio epidemic in the early 50s. (2, AM, p. 4)

As a child I had a mild case of polio during an epidemic. (2, AM, p. 5)

There was strong agreement among some town hall participants that all persons have a right to appropriate care from a health care professional, and that this right to care does not disappear during an emergency like pandemic influenza.

I think yeah, that everyone has the right to care. (2, AM, p.15)

Miss A is human and Mr. M is human and they all deserve the same level of respect and care. (3, AM, p.18)

I think the healthcare worker has to be taken care of, but I also think Mr. M does as well. They're both human. (3, AM, p.18)

However, while some participants also recognized that a right to care on the part of patients entails an obligation to treat on the part of health care professionals, others maintained that discharging the obligation to treat might have to be qualified in the context of an acute emergency that was overtaxing the healthcare system.

Well, yeah, we owe them something, but can we not provide some sort of care? Maybe we can't provide exactly what they need, but we can provide them comforts and something else, can't we? (3, AM, p.27)

Because even if we say to Mr. M, look you don't get the critical care bed... the responsibility is to provide as best a healthcare system as possible. Though [it] may not be ideal, but it still persists. (3, PM, p.15)

Another staring point shared by some participants was a reluctance to accept scarcity as a 'given'. Participants would sometimes propose a number of alternative solutions to facilitate access to care for anybody who needed care. Some participants questioned whether part of the solution to priority setting was merely redistributing medical resources creatively.

Strikes me that we only need to rethink how we use our resources and use them in more creative ways. (2, AM, p.15)

There are still other things to be done, other ways to allocate resources. (2, AM, p.24)

However, some participants suggested that the healthcare systems in their provinces were already in crisis and that a pandemic influenza would only serve to exacerbate this situation, i.e. that the healthcare system is 'broken' prior to an emergency.

There's never enough beds available in the hospitals for care. It's pretty much known. That's my gut reaction is that I get angry with the political cuts. (3, AM, p.4)

What happens to me? Like I don't even have a doctor to go to, so we don't even have doctors to go to right now, let alone if a pandemic hits this province, I think we are, yeah, screwed basically. (3, AM, p.10)

I have no faith in our system right now at all that we could handle a pandemic situation. (3, AM, p.10)

Some participants seemed to believe that certain members of society would receive preferential treatment and an unfair portion of resources during a pandemic. For example, some participants felt that in a time of crisis, healthcare workers would inevitably give priority to each other over non-worker patients and care for themselves or their own patients first.

What I'm concerned with is how things really work in the real world, and as I understand it, the good ol' boys network, they got their beds.... If you have a GP [family doctor] who is well entrenched in a [healthcare] network, you get better care. It's really common knowledge, so I want that on the table. Now, what that indicates is a lack of equity, okay. (2, AM, p.18)

You know healthcare workers [look] after healthcare workers. (3, PM, p.6)

Because she's [the nurse] got friends there, that people are going to slip her into the bed. (2, PM, p.12)

Some participants took certain values as 'givens', such that these values would not only constitute criteria to guide allocation (see the following subsection on *Criteria*), but also underpin thinking about priority setting as a whole. For example, some participants felt that maximizing the use of healthcare resources was the moral starting point upon which further priority setting decisions should be made, despite some of the negative repercussions that could follow this principle.

People are going to die and we have to try to save [the most people], do the best good for the most people. (1, AM, p.13)

So that when you're viewed as cold, heartless by the family, by other people, you can back up a step and say hang on a second, it may sound like that but we've gone through and realized that we're doing the most for the most people and we've had to use a very terrible set of criteria. (1, AM, p.32)

Others felt that those members of society in a position of power would necessarily get preferential treatment by virtue of their social positions.

I think it is not how much money you have, it's who you know. (3, PM, p.6)

These starting points shaped what values should guide priority setting criteria.

### Criteria for setting priorities

Participants from across the three town halls all put forth criteria, or guiding principles, that they felt needed to be considered and balanced when making priority setting decisions during a pandemic influenza. We have characterized these as substantive and procedural criteria. By 'substantive criteria', we mean general principles that participants felt ought to be considered when making priority setting decisions, which were often derived from their underlying moral assumptions. For example, some town hall participants tended to value utility, or "*do the best good for the most people" (1, AM, p.13)*, above other principles. Some participants felt that while maximizing the use of resources was important, other values need to be considered, including equitable treatment for all persons, "*human rights" (3, AM, p.6)*, and the "*dignity" (3, AM, p.9) *of all persons. Other substantive criteria included need (i.e. treat those who are in the greatest need of medical attention) and survivability (i.e. treat those who are most likely to survive medical treatment). By 'procedural criteria' we understand principles that would help decision-makers make decisions in real-time, e.g., first-come-first-serve (i.e. treating people based on the order in which they arrive at the hospital).

There was considerable discussion about whether resources should be allocated on the basis of societal contributions, i.e. "*not so much [social] status, but value [to] society, immediate value [to] society" (1, AM, p.17)*. For example, participants debated whether politicians deserved special treatment due to their roles in society.

You don't need a mayor - like you need a mayor for [the] long term, but day-to-day, if the mayor died, nobody would care for two years. (1, AM, p.16)

Another example is whether priority should be given to parents because of their caregiver role.

No, it makes a difference to me - there's three, possibly four people who are relying on that person. Not only is [he] the 'one of the bread winners', because we realize both parents have to work, but also as a family unit and how that would impact, I think, on the ages of the children. (1, AM, p.24)

On the basis of a person's value to society, most participants felt that the nurse, Ms. A, deserved to be prioritized over the non-healthcare worker patient, Mr. M. The justification for this prioritization differed among participants. Some participants felt that it was in society's best interest to give healthcare workers priority access to health services in order to ensure a strong and sustainable health infrastructure throughout a pandemic. A similar view held that not giving priority to healthcare workers might undermine the effectiveness of the overall health workforce:

If this nursing staff doesn't receive priority care over Mr. M. it will demoralize the rest of the nursing staff. We're out of here, we're gone, everybody can die, because we're not putting our lives in danger. (2, AM, p.20)

If the healthcare worker is not looked after, what's going to happen to the other healthcare workers when they see this? If they're not going to look after her, they're not going to look after me. I'll put on my coat and hat and get out of here, leave the hospital, or finish my shift and book off sick the next four days or three days, or whatever. (3, AM, p.17-18)

Other participants felt that society has a reciprocal duty to healthcare workers because of the risks they take to care for others.

[A nurse] has endangered their life in giving a public service and on that basis, [she] becomes a priority. (2, AM, p.21)

She accepted that she had an obligation to society and I think as a society we need to accept that we have a duty to her as well. (2, PM, p.4)

It should be noted, however, that not all participants gave priority to healthcare workers over members of the general public.

Is it really too much to ask that everyone be treated as equals the whole way through?.... How do you really place a value on somebody? Shouldn't everybody be on the same level? (3, PM, p.11)

Participants, therefore, discussed and weighed the pros and cons of different procedural and substantive criteria by which to set priorities during a pandemic influenza. Of note, special attention was given to whether someone's social status or employment in the healthcare sector granted a person priority access to scarce resources.

### Pre-crisis planning

Across all three town halls, participants felt that governments, health agencies, and hospitals needed to have plans in place to guide decision-making prior to the occurrence of pandemic influenza.

You need to know what your priorities and policies are before you walk into this [crisis situation]. (2, AM, p.15)

I really truly think that in times of a pandemic, we'll have to come up with a plan. (3, PM, p.7)

There must be guidelines at the ER. (3, PM, p.6)

Part of the justification for requiring planning ahead of a crisis was to avoid overburdening decision-makers with the brunt of ethical responsibility associated to making difficult, often life-and-death, decisions. Participants commonly described having to make priority setting decisions as difficult, no-win situations, and as psychologically burdensome.

It sucks, but people are going to die.... [My] personal feeling on it is 'tough luck'. (1, AM, p.13)

We were talking about just how difficult it is to ask those [priority setting] questions. (3, AM, p.2)

Who makes the decision to where Mr. M is placed? That would be a tough job. (3, AM, p.9)

I can take charge of situations if I need to, there's no doubt about that and I know most people can, but especially if they know what the rules are, here we go, we've got a plan and everybody's got that same plan, then maybe we can implement it when we need to. (3, AM, p.10)

Another reason put forth for planning was to avoid conflict during a crisis regarding who would be making decisions and what values would guide priority setting.

We have to have a plan and if we have a plan and some guidelines, then hopefully they will see us through in the most ethical way possible. (3, PM, p.11)

To me this is what this is all about, is planning. It's about looking ahead, it's about having something to [help you decide], so that you don't have to be forced into those situations where you're subjective and emotional and all those kind of things, so the priorities have to be made in advance, while everybody is thinking clearly. (2, PM, p.28)

You were saying the poor decision maker and you're right, to make those decisions, I would not want to. Hopefully it's this sort of thinking beforehand that would facilitate and should at least make the responsibilities shared. (3, PM, p.5)

Participants also felt that having plans before the onset of a pandemic influenza might help ensure a fair and transparent decision making process.

I'm afraid that unless the priorities that are established in the planning stages, like in advance of the pandemic, unless they're really transparent, that a pecking order will result. (2, PM, p.12)

I think they [decisions] have to be made in advance, they have to be made now, they need to be pragmatic, they need to be transparent. (2, PM, p.27)

[The public] should know going into [the] hospital who takes priority. (3, PM, p.15)

At the same time, some participants noted that it is difficult to predict what will occur during a public health crisis, thereby making it difficult to plan for these situations. Others believed that plans would likely be ignored during a crisis because of the inherent stress of the situation.

I don't know if there's anything you can pre-plan, but that's what I suspect, because often in crisis situations, people will think - their heads just start to think, right, and you come up with solutions. (3, AM, p.5)

Even if there's a plan, I don't have the faith to believe that yes, we have a plan and this is what we're going to do. (3, AM, p.10)

Faced with adversity and in that situation, plans seem to go out the window. (3, AM, p.11)

Overall, participants generally believed that planning for a pandemic influenza was prudent and might make in-crisis decision making easier for those who ultimately will have to make difficult decisions.

### In-crisis decision-making

Despite the support for having guidelines in advance of a pandemic influenza, participants acknowledged that difficult decisions would need to be made in the moment during a crisis.

So decisions have to be made and people have to make them in a time of emergency and the powers have to be given to those decision makers to make those decisions. (3, PM, p.23)

It seems to me that things probably get pretty hairy, pretty fast and decisions have to be made quite quickly and there's not really going to be any time to defer on a case. (2, PM, p.26)

Across the three town halls, participants often expressed that decisions should be made by a committee in order to avoid having any one solitary individual shoulder the entire burden of making potentially life-and-death decisions during a pandemic influenza outbreak.

More of a committee type and then you get a consensus or you get a quorum or something and you act upon it in that way. (3, PM, p.13)

In an ideal world, yeah, you'd have more than one person making that decision. (3, PM, p.19)

Most participants also believed that a variety of perspectives and persons should be involved in the decision-making process, not merely physicians. Although there was no agreement as to the composition of these decision-making committees, participants felt that they should include physicians, nurses, other healthcare workers, lawyers, patient advocates, and ethicists.

I would say that in the hospital, the healthcare workers, doctors, nurses, emergency responders, everybody, like whoever is involved in making decisions, the people with the most expertise have to be the people who make a decision in a crisis situation. (3, PM, p.19)

I think there should be nursing included because they spend [the most] time with the patients. (2, PM, p.26)

But we don't want just necessarily healthcare, you want the opinions of an ethical kind of, you know, a person who has some kind of staff training. Just a bit more variety than just a doctor or a nurse. (2, PM, p.26)

I'm a little bit confused as to who should actually make the decisions. (2, PM, p. 27)

However, a minority of participants also stated that physicians should have the final say in allocation decisions, especially if consensus was not possible or forthcoming.

It has to be his decision, the doctor in charge, I would think. If they can consult with the family a little bit, if there's time, but otherwise I'd leave it to the doctor. (3, PM, p.19)

Leave it up to the healthcare workers, you know, the doctors. (3, PM, p.21)

The person in charge of the ER would have to make the decision of Mr. M getting a room. (3, PM, p.15)

There appeared to be a sense among some participants that the normative capabilities required to make difficult decisions do not rest with only one type of person or, indeed, solely with professionals or experts.

I'm sorry, but your best doctor and your best lawyer do not know anything more about ethics than my mom, and that's the way it is. (2, PM, p.30)

Some participants also raised the possibility of patients or family members being involved with the decision-making regarding who would get scarce resources (e.g. "*What about the family members or family?" - SJ, PM, p.16) *although it was readily acknowledged that this would be more of an advocacy role rather than any actual decision-making power. One participant also contemplated having an appeals mechanism for patients and their families regarding priority setting decisions.

I thought guidelines would be good and yes, they should be followed as long as the patient or whomever would have the ability to appeal if they didn't like the outcome, but then I'm thinking in my head again, there's no time. How do you appeal? (3, PM, p.20)

Despite the need for pre-crisis planning, most participants felt that difficult priority setting decisions would need to be made by committees due to the contextual factors that arise during specific cases.

### Need for public deliberation and input

Participants generally felt that pre-pandemic planning should be done in consultation with the public.

I think forums such as these are a really good way of getting some input in from the public. (2, PM, p.27)

Well, I think people are going to buy into it [i.e. decisions] more when it's more bottom-up than top-down. (2, PM, p.33)

[Guidelines] have to be created and the public has to be aware of them.... And have input. (3, PM, p.16)

In creating the guidelines, we all should be involved. (3, PM, p.17)

In particular, one participant noted that the public needs to be educated regarding the complexity of making priority setting decisions.

Education has to be part of the equation. It's very difficult. Education of, well, of us, right? It's really hard to wrap your head around the idea of the kinds of demands that are going to be made, the conditions that are going to exist. (2, PM, p.26)

However, some participants expressed doubt as to whether public consultation and input would actually be used to guide decision-making regarding priority setting or rather used as 'window-dressing'.

Democratically approved policies, criteria, out in front and to me, I mean I think that criteria [have] been established and policies made already and they have not been made by us and by us, I believe us means the [the public]. (2, PM, p.26)

This is interesting research but will this go anywhere, you know? But for [this province], I mean, wouldn't it be nice that whoever does come up with the guidelines to follow during the pandemic, they let us know and they give us some sort of avenue to give our feedback? (3, PM, p.16)

### Deliberative struggle

Participants across the town halls demonstrated what might be described as 'deliberative struggles' in their attempts to address the ethical quandaries posed by the priority setting scenario. Often, this was expressed as uncertainty about which ethical values to prioritize over others, i.e. how to balance different ethical values.

[That] it even needs to be considered that one human deserves more consideration than another [when allocating resources]. That boggles my mind. I just, I, I keep going over it in my head. I don't know. Pretty compelling. (3, AM, p.25)

Well after all of this discussion, my decision is somewhat clouded. (2, PM, p.22)

Some participants expressed distress about being asked to generate reasons or justifications for allocating scarce medical resources.

You have this sea of death around you, you can go home and say there's not enough whisky I can drink to erase this whole concept. (1, AM, p.33)

[In jest, given the ethical difficulty] Get me out of here! (2, PM, p.28)

Well, it's a very overwhelming situation. That's how I look at it and say very overwhelming. (3, AM, p.5)

One participant expressed her confusion reconsidered earlier pronouncements.

What surprised me is that I've gone through this exercise and how clear I may have been at one point and how completely confused I am now.... I question you know, the strength of my earlier decisions, you know. (2, PM, p.27-28)

Even for those participants who did not change their opinions as the day progressed, there was evidence of their appreciation for the difficulty of making priority setting decisions in light of scarce resources.

Well, it didn't change anything for me, but you have to consider this new piece of information, he has aging parents at home [but] that doesn't compel me to make a different decision... (3, PM, p.7)

Everybody is valuable in their own world, in their own way and it's going to be tricky, I know that, but right now, no, it [my decision] has not changed. (2, PM, p.9)

It doesn't change anything for me. I certainly feel more compassionate for Mr. M. He seems to be a caring individual, taking care of his parents so I value him more highly than just an individual that has had a bicycle accident... (2, PM, p.14)

In addition, the deliberative struggle was not just internal to individual participants, but also occurred between participants. Certain passages of the transcript suggest that participants were comfortable expressing differences of opinions with regard to the values that should guide priority setting.

*Participant X: So she accepted that she had an obligation to society and I think as a society we need to accept that we have a duty to her as well, and I think that's the flip side of that coin, which we cannot avoid*.

Participant Y: What percentage of her dollars went to build the hospital more than anybody else's?

*Participant X: That's not the same thing*.

*Participant Y: The hospitals are built with care of everybody, not certain groups*.

Participant X: No, they're not. But if we are in position where we are dependent upon people to make our lives possible and in terms of the healthcare worker, certainly serving sick people and serving sick people during a pandemic is incredibly risky. (2, PM, p. 4)

The willingness with which participants engaged in these deliberative struggles supports previous research on the ability of the general public to consider difficult and complex decisions about public policy

## Discussion

Based on the six themes identified above, we present four observations with important implications for governments and health policy makers with regard to planning for influenza pandemics and public health emergencies more broadly.

First, our analysis identified a notable level of cynicism among participants about the health system's current capacity to respond effectively to an influenza pandemic. The health system was described as already in crisis and barely able to meet existing demand, let alone the overwhelming demand of an influenza pandemic. Participants also articulated working assumptions about the behavioural responses of healthcare workers in a crisis. On the one hand, there were concerns that healthcare workers would 'protect their own' or avoid coming to work if other colleagues contracted the influenza virus. On the other hand, participants expressed considerable empathy toward the burden of moral responsibility healthcare workers are faced with making 'tough choices' about patients' lives. Finally, while participants recognized that standard of care might not be achievable in a pandemic influenza crisis, they emphasized that care was owed to all patients even if it were simply comfort measures. Understanding the public's 'starting points' may have important implications for policy makers in terms of governments being able to effectively engage, inform, and communicate with the public in a timely manner, especially during public health emergencies like an influenza pandemic. For example, communicating clearly the reasons why healthcare workers might be prioritized over non-healthcare workers for scarce resources during a pandemic (e.g. because they put their lives in danger by the nature of their work) would need to take into account that the public may be somewhat distrustful of healthcare workers (e.g. the finding that some participants believed that the healthcare system is already biased in favor of healthcare workers). Building trust may signal to the public that scarce resources are being valued for what they are, scare resources, and that these resources are not being 'wasted' frivolously.

Second, participants underscored the challenges of empirical uncertainty in ethically charged decision-making moments. While participants often sought more facts about the priority setting scenario as presented, when more facts were not available, participants explored alternate formulations of the priority setting scenario based on different empirical assumptions to test their positions, values, or conclusions. For example, the scenario did not specify how Ms. A (the nurse) had contracted the influenza virus. In all three town halls, participants considered explicitly whether it made a difference in their assessment of the relative claim of Ms A and Mr M for an ICU bed if Ms A had contracted the virus at work rather than in the community. Notably, this would often include deliberation on the social value of 'essential workers' in an influenza pandemic and the appropriateness of considering social value in allocating limited health resources. Participants recognized that in an influenza pandemic, policymakers and clinicians would be faced with making decisions with incomplete information. Given this uncertainty, participants placed great emphasis on the need for planning and agreement about decision-making principles and values to bridge these empirical gaps and ease the moral and psychological burden of decision-makers in the midst of a crisis. Our findings suggest a considerable appreciation among town hall participants of the inherent uncertainty of pandemic response. Clear communication by decision makers about the nature and extent of this uncertainty may make a greater contribution to building public trust than issuing unfounded assurances of certainty intended to allay public fears.

Third, when given time to deliberate about ethical quandaries, like priority setting in the context of influenza pandemics, participants demonstrated nuanced ethical reasoning. Participants struggled with regard to which values should guide priority setting decisions. Overall, the participants rejected the pragmatic constraints imposed by the assumption of extreme scarcity. While participants acknowledged that scarcity was a reality, the deliberation would often start with the assumption that 'something could be done' and focus on alternate practical solutions to bridge the scarcity gap (e.g. whether healthcare staff outside the ICU could be taught the basic necessities of the job; whether resources could be reallocated from different departments of the hospital to ease the ICU's burden; whether the lay community might be able to fill the gap). When 'nothing could be done' to bridge the scarcity gap, participants articulated the struggle in moral terms about what values and criteria should guide priority setting decisions. Participants acknowledged and understood the inherent ethical difficulties in having to make allocation decisions under duress, uncertainty, and time-constraints. Participants reinforced the importance of planning not only as a means for easing the moral and psychological burden of decision-makers, but also as a means of building a broad consensus and ensuring transparency about how priority setting decisions would be made before the constraints of a crisis created an impediment to deliberation and engagement.

Finally, the participants proposed a number of ethical values and criteria that they perceived to be relevant to allocating ICU beds during an influenza pandemic; this is consistent with other empirical studies examining public perspectives on priority setting in a pandemic. For example, Ritvo and colleagues conducted a national telephone survey of Canadian residents, in which participants were asked to identify who should have priority for access to scarce hospital resources [29]. Participants attributed high priority to children, healthcare workers infected while serving patients, the sickest patients, and adults with dependents, which suggests a number of prioritization principles were at work (e.g., fair innings, reciprocity, compassion). Much like Ritvo et al's study and our Canadian town halls, Vawter and colleagues, who conducted town hall meetings with Minnesota residents, found that participants also elected to ration ventilators on the basis of varying values (e.g. solidarity and mutual responsibility) [25]. Moreover, the Minnesota town hall participants contended that life expectancy and socio-economic status *should not *be considerations in allocating ventilators during an influenza pandemic. One should note that our empirical findings (along with those of Ritvo et al. and Vawter et al.) seem to be at odds with the majority of theoretical papers that have espoused utility, i.e., maximizing good outcomes with available resources, as the overarching ethical consideration in allocating ICU beds during influenza pandemics,[7-12] including those which argue against giving priority to health care workers [14]. The limits of utility-based theories of emergency ICU bed allocation have also been critiqued in the theoretical literature, especially on the basis of equity with regard to criteria that may disadvantage existing vulnerable populations [6,13].

A possible limitation of our findings is their generalizability to other health systems or other public health outbreaks and challenges. As a qualitative study, our goal was not generalizability. However, we expect that readers in other health systems or faced with other public health challenges may see themselves in our findings. For example, the balancing of different ethical criteria and the need for public input have been found in other studies on public engagement in priority setting in healthcare beyond the context of pandemic planning [30-32]. Moreover, access to ICU resources is a perennial challenge during seasonal influenza outbreaks, for which the importance of pre-planning, public accountability, and transparency are relevant factors in establishing and maintaining public trust. Another question that one may arise is the applicability and usefulness of qualitative data in the context of healthcare policy discussions. The CanPREP town halls were part of a larger research project looking at pandemic ethics, which included the previously mentioned telephone survey by Ritvo and colleagues [29]. While the national telephone survey provided a breadth of information about Canadians' perspectives on resource allocation and other ethical issues in a pandemic, the town hall deliberations provided insight into the underlying justification or rationale for the survey findings. For example, although the survey indicated a preference for healthcare workers having priority access to for scarce medical resources, it is through the town hall deliberations that we were able to identify intrinsic (e.g. 'society owes them') and instrumental (e.g. 'society needs happy healthcare workers') reasons for this preference whilst also elucidating the ethical and practical complexities entailed in acting on this preference. Finally, our findings may not be broadly representative of Canadians' perspectives on priority setting of intensive care resources in an influenza pandemic. This is due in due in part to the limitations of our sampling strategy, which was not designed to achieve representativeness; it may also be due to the pragmatic demands of participating in a full-day town hall, which may not have been feasible for some interested individuals due to employment, child care, or other constraints. Notwithstanding these limitations, the town hall participants were remarkably diverse in their experience and provided a rich narrative to enhance our understanding of the survey findings and shed light on how some Canadians might address the ethical issues raised by an influenza pandemic.

## Conclusion

In light of the preexisting theoretical literature examining how priorities should be set regarding scarce medical resources during a pandemic influenza, this qualitative study provides the first set of results regarding the values that the Canadian public holds regarding these important and challenging ethical issues. Our results indicate that our town hall participants were not only concerned with the values or criteria that should guide priority setting decision-making, but also with *who *and *how *those decisions should be made. We suggest that taking seriously the ethical and empirical starting points of the public, along with their acknowledgment of the role of uncertainty in decision-making in times of duress, would contribute to building public trust in the healthcare system's ability to set priorities in a just and fair manner during public health emergencies.

## Competing interests

The authors declare that they have no competing interests.

## Authors' contributions

DS participated in the design of the study and data collection, analysis, and interpretation, and drafted the paper. JG participated in the conception and design of the study and data analysis and interpretation, and co-drafted the paper. AR participated in the conception and design of the study and data collection, analysis, and interpretation, and contributed to drafting the paper. CB participated in the design of the study and data collection, analysis, and interpretation, and contributed to drafting the paper. SS participated in data collection, analysis, and interpretation, and contributed to drafting the paper. LM participated in data analysis and interpretation, and contributed to drafting the paper. MS participated in data analysis and interpretation, and contributed to drafting the paper. All authors read and approved the final manuscript.

## Pre-publication history

The pre-publication history for this paper can be accessed here:

http://www.biomedcentral.com/1471-2458/12/241/prepub

## Supplementary Material

Additional file 1 **Priority Setting Scenario**. Click here for file
