# Supplementary material for: Priority setting of ICU resources in an influenza pandemic: a qualitative study of the Canadian public's perspectives
Source: BMC Public Health. 2012 Mar 26;12:241. doi: 10.1186/1471-2458-12-241 (PMC3331804; doi:10.1186/1471-2458-12-241)
Supplement: Additional file 1 — Priority Setting Scenario. [file 1471-2458-12-241-S1.DOC]

Additional file 1 – Priority Setting Scenario

**Initial Scenario**

The Vancouver General Hospital is a major trauma centre with a large emergency department and intensive care unit (ICU). During a pandemic influenza crisis outbreak in Vancouver,  the  ICU is filled to capacity with patients suffering from life-threatening medical conditions including complications from influenza like bacterial pneumonia. The emergency department calls the ICU seeking to admit Mr M, who was brought to the emergency room with a severe but potentially reversible brain injury after a bicycling accident. One alternative is to move one of the current ICU patients to a medical unit in order to make room for Mr M. However, the ICU staff reports that all of their patients need ventilator support and there are no other ventilated beds available in the hospital. Another alternative is to send Mr M to one of the other units of the hospital. However, given Mr M's injuries, it is clear that this would overtax the clinical capabilities of the health care staff who are not trained in dealing with ventilation on those units, who are already struggling to care for patients. The final alternative is to transfer Mr M to another health care facility. However, the influenza pandemic has overwhelmed all hospitals in the region and there are no available ICU beds anywhere else.

**First Set of Questions**

1. What are your initial responses to this situation?  What is your gut reaction?
2. What are the most important considerations in this scenario? Which ones are the most relevant?
3. What are the features of this case that you find the most compelling?

**Scenario Continued (Reveal 1)**

An ICU patient passes away. There is now a bed available in the ICU for Mr. M. Just as his transfer is about to be made, an ICU nurse named Ms. A is admitted with severe difficulty breathing. It is determined that she has been infected with the influenza virus, which she may have contracted while caring for patients in the hospital's ICU. She needs immediate ventilation support, which is only available in the ICU bed designated for, but not yet occupied by, Mr. M.

**Second Set of Questions**

4. Have your initial responses to the situation changed in light of this information?

5. Are there any considerations that you find significant in this latest piece of information?

6. Is there anything compelling about this development?

**Scenario Continued (Reveal 2)**

It comes to light that Mr. M has aging parents at home who rely on him for help with their activities of daily living.  Ms. A is a longstanding employee of the hospital and is well respected by her peers at the hospital.

**Third Set of Questions**

7. Have your responses to the situation changed in light of this information?

8. Are there any considerations that you find significant in this latest piece of information?

9. Is there anything compelling about this development?

10. Do these personal details about the two patient's lives affect your perspective on this case?

**Final Questions**

11. Who should be given the ICU bed: Mr. M or Ms. A?

12. Who should make these kinds of decisions?

13. How should these kinds of decisions be made?

14. In the absence of consensus, how should these decisions be made?
